# Rapid Identification and Antimicrobial Susceptibility Testing for Urinary Tract Pathogens by Direct Analysis of Urine Samples Using a MALDI-TOF MS-Based Combined Protocol

**DOI:** 10.3389/fmicb.2019.01182

**Published:** 2019-06-05

**Authors:** Wei Li, Enhua Sun, Ying Wang, Hongwei Pan, Yi Zhang, Yong Li, Xin Zhang, Chen Li, Lutao Du, Chuanxin Wang

**Affiliations:** ^1^Department of Clinical Laboratory, Qilu Hospital of Shandong University, Jinan, China; ^2^Department of Clinical Laboratory, The Second Hospital of Shandong University, Jinan, China

**Keywords:** bacterial identification, antimicrobial susceptibility, MALDI-TOF MS, urinary tract infections, rapid diagnosis, urine

## Abstract

Usually, 18–48 h are needed for the identification of microbial pathogens causing urinary tract infections (UTIs) by urine culture. Moreover, antimicrobial susceptibility testing (AST) takes an additional 18–24 h. Rapid identification and AST of the pathogens allow fast and precise treatment. The objective of this study was to shorten the time of diagnosis of UTIs by combining pathogen screening through flow cytometry, microbial identification by matrix-assisted laser desorption ionisation time-of-flight mass spectrometry (MALDI-TOF MS), and AST using the VITEK 2 system for the direct analysis of urine samples. We analyzed 1,638 urine samples from patients with suspected UTIs submitted to the microbiology laboratory for culture. Each urine sample had an approximate volume of 30 mL and was divided into three aliquots. Urine processing included differential centrifugation and two washes to enrich the bacterial fraction for direct MALDI-TOF MS and direct AST. From a total of 1,638 urine samples, 307 were found to be positive through UF-1000i screening. Among them, 265 had significant growth of a single-microorganism. Direct identification was obtained in 229 (86.42%) out of these 265 samples, and no pathogens were misidentified. Moreover, species-level identification was obtained in 163 (88.59%) out of the 184 samples with Gram-negative bacteria, and 27 (38.03%) out of the 71 samples with Gram-positive bacteria. VITEK 2 AST was performed for 117 samples with a single-microorganism. *Enterobacteriaceae* data showed an agreement rate of antimicrobial categories of 94.83% (1,229/1,296), with minor, major, and very major error rates of 4.17% (54/1,296), 0.92% (12/1,296), and 0.08% (1/1,296), respectively. For *Enterococcus* spp., the overall categorical agreement was 92.94% (158/170), with a minor error rate of 2.94% (5/170) and major error rate of 4.12% (7/170). The turnaround time of this combined protocol to diagnose UTIs was 1 h for pathogen identification and 6–24 h for AST; noteworthily, only 6–8 h are needed for AST of *Enterobacteriaceae* using the VITEK 2 system. Overall, our findings show that the combination of flow cytometry, MALDI-TOF MS, and VITEK 2 provided a direct, rapid, and reliable identification and AST method for assessing urine samples, especially for Gram-negative bacterial infections.

## Introduction

Urinary tract infections (UTIs) are among the most common community- and hospital-associated bacterial infections; affecting about 150 million persons worldwide each year (Flores-Mireles et al., 2015; McLellan and Hunstad, 2016). Uropathogenic *Escherichia coli* (UPEC) is the most common causative agent of UTIs, it is responsible for more than 80% of the community-acquired UTIs (Terlizzi et al., 2017). Other pathogens include *Klebsiella pneumoniae*, *Proteus mirabilis*, *Staphylococcus saprophyticus*, *Enterococcus faecalis*, group B *Streptococcus* (GBS), *Pseudomonas aeruginosa*, *Staphylococcus aureus*, and *Candida* spp., which are particularly relevant as hospital-acquired and catheter-associated infectious agents (Flores-Mireles et al., 2015; McLellan and Hunstad, 2016). Urine culture remains the gold standard method to identify UTI pathogens, but it is time consuming. In conventional laboratory UTI diagnostics, identification of pathogens takes 18–48 h, and antimicrobial susceptibility testing (AST) needs an additional 18–24 h. Moreover, even though traditional screening methods, such as Gram-staining, flow cytometry, and urine dipstick testing, allow the rapid exclusion of negative samples and a primary identification of pathogens in positive samples (Íñigo et al., 2016), information obtained from these screening methods is insufficient for making decisions regarding antibiotic treatment (Davenport et al., 2017). Patients may be empirically treated with antibiotics (sulfamethoxazole/trimethoprim, nitrofurantoin, or ciprofloxacin), and these may be changed if the results from the AST show that it is needed. However, an inappropriate use of antibiotics may delay effective treatment and contribute to the rise of multidrug-resistant organisms. Therefore, a novel methodology is needed for both timely identification of pathogens and AST.

In the past years, matrix-assisted laser desorption ionisation–time of flight mass spectrometry (MALDI-TOF MS) has been considered as a rapid and trustable technique for identifying microorganisms from culture plates, positive blood cultures, and different clinical samples, such as urine (Ferreira et al., 2011; Clark et al., 2013). Previous studies have shown that urine itself can be used for direct identification by MALDI-TOF MS; besides, several studies have been carried out to improve sample processing and MALDI-TOF MS identification capacity (Ferreira et al., 2010; Wang et al., 2013; Burillo et al., 2014; Íñigo et al., 2016; Zboromyrska et al., 2016; Huang et al., 2017; Zboromyrska et al., 2018). However, these studies have focused on direct identification only, while not having addressed direct AST from urine, especially to accurately determine minimum inhibitory concentrations (MICs). Furthermore, AST is the most important step for the determination of a proper antibiotic treatment and a direct AST method could greatly shorten the time to obtain AST results.

In this study, we investigated a whole MALDI-TOF MS-based workflow to shorten the time of pathogenic diagnosis and effective treatment of UTIs by combining flow cytometry for screening, MALDI-TOF MS for microbial identification, and the VITEK 2 system for AST of urine samples directly.

## Materials and Methods

### Clinical Samples and Study Design

**FIGURE 1:**
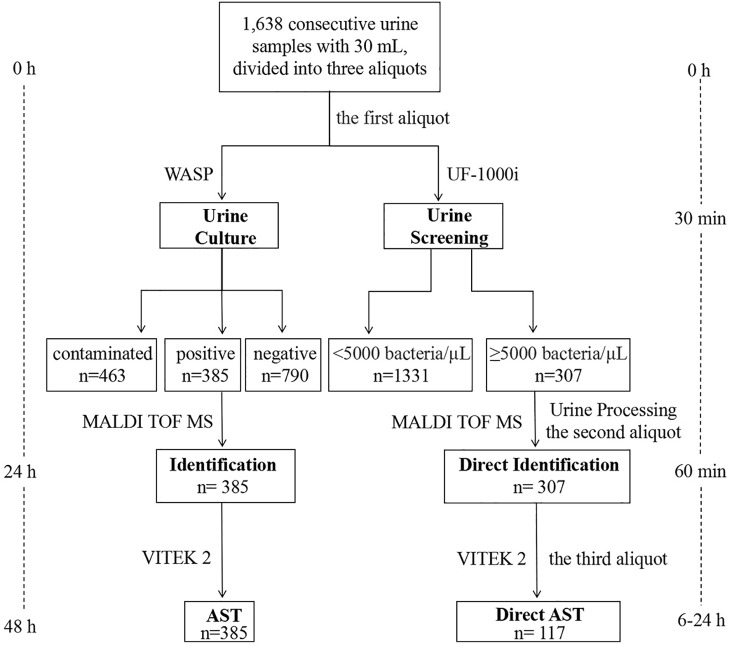
Workflow chart of MALDI-TOF MS-based combined protocol (right) versus conventional culture-dependent protocol (left), showing the turnaround time from arrival of the urine sample in the microbiology laboratory until identification and AST.

A total of 1,638 consecutive urine samples without any chemical preservatives were submitted for culture to the Clinical Microbiology Laboratory, Department of Clinical Laboratory, Qilu Hospital of Shandong University (Jinan, China) during September and December 2018, from inpatients and outpatients. As shown in Figure 1, each urine sample had an approximate volume of 30 mL, that was divided into three aliquots using 15 mL sterile centrifuge tubes. The first aliquot was used for culture by a Walk Away Specimen Processor (WASP, Copan, Brescia, Italy), and then for a UF-1000i screening (Sysmex, Kobe, Japan). If the result of bacterial counts was ≥5,000 bacteria/μL, the other two aliquots were used to prepare bacterial pellets. The first one was used for direct identification by the MALDI-TOF MS (Bruker Daltonik GmbH, Bremen, Germany). Once with a reliable identification, the second bacterial pellet was used for direct AST by the VITEK 2 system (bioMérieux, Marcy l’Étoile, France).

### Conventional Urine Culture for Pathogen Identification and AST

In our laboratory, all the urine samples submitted for culture were processed by a fully automated plating and streaking instrument, the Copan WASP. The first 10 mL urine aliquot was plated for culturing by the WASP processor using 10 μL sample loops on blood agar and MacConkey agar plates. Plates were incubated aerobically for 24–48 h at 37°C. Urine samples were classified as positive with a UTI pathogen growth >10^4^/mL and a positive leukocyte esterase test result. The recovery of 2 different bacterial species with a similar growth rate, >10^4^/mL, and a positive leukocyte esterase test result was also considered positive. When the bacterial load of one species was >10^4^/mL and lower for the other, the species with a predominant growth was considered clinically significant. However, samples with more than two microorganisms were considered contaminated. Single colonies grown on blood agar plates were counted and the bacterial concentration (CFU/mL) was calculated for each urine sample. Finally, identification was performed by MALDI-TOF MS. Strains with non-reliable identification (NRI) results were confirmed by the VITEK 2 Compact system. The AST was performed on the VITEK 2 Compact system. VITEK cards AST-GN13, AST-GN09, and AST-GP67 were used for *Enterobacteriaceae*, non-fermenting Gram-negative bacilli, and *Staphylococci*/*Enterococci*, respectively. All protocols were performed according to the manufacturers’ instructions. The ATCC strains *Escherichia coli* 25922, *Pseudomonas aeruginosa* 27853, *Enterococcus faecalis* 29212, and *Staphylococcus aureus* 29213 were used as quality controls of the VITEK 2 Compact System and AST cards to ensure the credibility of AST results.

### Flow Cytometry

After WASP processing for culture, the first aliquot was screened for bacteria by using a UF-1000*i* flow cytometer (Sysmex, Kobe, Japan), according to the manufacturer’s instructions. Samples with bacterial counts of ≥5,000 bacteria/μL were considered as positive for further processing.

### Urine Processing and Preparation

The second and third aliquots were vortexed and centrifuged at 2,000 *g* for 1 min to remove leukocytes, cellular debris and mucus. The resulting supernatant was centrifuged once again at 10,000 *g* for 5 min, separated from the pellet, and discarded. Bacterial cell pellets were resuspended and washed in 1 mL of 0.45% saline solution (bioMérieux, Marcy l’Étoile, France) and transferred into 1.5 mL microcentrifuge tubes. Once again, bacterial suspensions were pelleted by centrifugation, 10,000 *g* for 2 min, and washed.

### Direct MALDI-TOF MS Identification

The bacterial cell pellet of the second aliquot was dissolved in 50 μL of formic acid 70% by vortexing; then, 50 μL of pure acetonitrile was incorporated into the solution, vortexed, and centrifuged (10,000 *g* for 2 min) for the following direct MALDI-TOF MS identification assay. In brief, 1 μL supernatant was spotted onto a target plate, and 1 μL of an α-cyano-4-hydroxycinnamic acid (HCCA) matrix solution (Bruker Daltonik GmbH, Bremen, Germany) was added and air dried at room temperature. Identifications were performed by the Bruker microflex MALDI-TOF MS system using the MALDI Biotyper 3.0 RTC database (Bruker Daltonik GmbH, Bremen, Germany). According to the manufacturer, a score of ≥2.0 indicates reliable identification at the species level, while a score between 1.7 and 1.99 indicates a reliable identification at the genus level, and a score below 1.7 indicates a NRI. Thus, these were the criteria used when comparing the results of the direct MALDI-TOF MS identification assay with those from the conventional culture-dependent identification for the evaluation of direct identification performance.

### Direct VITEK 2 AST

Once a reliable direct identification by MALDI-TOF MS was achieved, the pellet of the third aliquot was diluted in a saline solution 0.45% and its density adjusted to 0.5 McFarland turbidity standard. Then, it was used for a direct AST by the VITEK-2 Compact System. All protocols were performed according to the manufacturer’s instructions. VITEK cards AST-GN13, AST-GN09, AST-GP67 were used for *Enterobacteriaceae*, non-fermenting Gram-negative bacilli, and *Staphylococci*/*Enterococci*, respectively.

### Data Analysis

To compare the results from direct AST with those of conventional culture-dependent AST, the MIC values obtained by both methods were reclassified into clinical categories (susceptible, intermediate, and resistant) according to the Clinical and Laboratory Standards Institute’s (CLSI) clinical breakpoints (Clinical and Laboratory Standards Institute [CLSI], 2018b); the categorical agreement rates as well as the error rates were calculated as recommended by the CLSI M23Ed5 document (Clinical and Laboratory Standards Institute [CLSI], 2018a). For evaluation purposes, we defined the conventional culture-dependent method as our “gold standard” method. We considered that we had a categorical agreement if the results from both AST methods were within the same susceptibility category. The category errors were defined as follows: minor error, bacterial isolates showing an intermediate result by direct AST but either resistant or susceptible by conventional culture-dependent AST, or *vice-versa*; major error, strains shown to be susceptible by conventional culture-dependent AST but resistant by direct AST (false resistance); very major error, strains were shown to be resistant by conventional culture-dependent AST, but susceptible by direct AST (false susceptibility).

## Results

### UF-1000i Flow Cytometry Analysis

For this study, 1,638 urine samples submitted to our clinical microbiology laboratory with sufficient volumes were screened for bacteria through a UF-1000i cytometer. Based on a previously established bacterial counts cut-off value of ≥5,000 bacteria/μL (Zboromyrska et al., 2016, 2018), 307 (18.74%) out of 1,638 samples were positive and used for further direct identification. Among these positive samples, 265 had a significant growth of a single microorganism by urine culture, 16 had a significant growth of two microorganisms, 22 were contaminated, and 4 were negative (Table 1). Among the 1,331 urine samples with negative bacterial screening results, 104 were positive by urine culture with a significant growth, 241 were contaminated, and 986 were negative (Table 1). In this study, the omission ratio of urine samples for further direct identification was 6.35% (104/1,638) when the bacterial counts cut-off value was set to ≥5,000 bacteria/μL. From those samples with a concentration <5,000 bacteria/μL, 98 had a significant growth of a single microorganism, including 65 samples with *Enterobacteriaceae*, 16 with *Enterococcus* spp., 5 with non-fermenting Gram-negative bacilli, 5 with *Staphylococcus* spp., and 7 with *Candida*. Additionally, 6 samples had a significant growth of two microorganisms, including 2 samples with *Escherichia coli* + *Klebsiella pneumoniae*, 1 sample with *Escherichia coli* + *Candida glabrata*, 1 sample with *Candida tropicalis* + *Enterococcus faecium*, 1 sample with *Enterococcus avium* + *Staphylococcus haemolyticus*, and 1 sample with *Klebsiella pneumoniae* + *Enterococcus faecalis*. Bacterial counts in these 104 urine samples were between 500 and 5,000 bacteria/μL.

**Table 1:** Correlation between UF1000i bacterial count and culture results in 1638 urine samples.

	UF1000i bacterial count (bacteria/μL)	
Culture results	≥5,000 *n* (%)	<5,000 *n* (%)
Positive	281 (91.53)	104 (7.81)
Single-microorganism	265 (86.32)	98 (7.36)
Two-microorganism	16 (5.21)	6 (0.45)
Contaminated	22 (7.17)	241 (18.11)
Negative	4 (1.3)	986 (74.08)
Total	307 (100)	1,331 (100)

### MALDI-TOF MS Direct Identification Results

In this study, 307 out of 1,638 bacterial pellets from urine samples were directly identified by the MALDI-TOF MS. The direct identification results of 265 single*-*microorganism samples were compared with those from the conventional culture-dependent method. As shown in Table 2, reliable direct identification was obtained in 229 (86.42%) out of 265 single-microorganism samples, while 36 (13.58%) samples were NRI (*n* = 24) or with no peaks (NP, *n* = 12). No bacteria were misidentified and species-level identification (score ≥ 2.0) was obtained for 190 out of these 229 samples. Direct identification showed that the dominating microorganisms were *Escherichia coli* (99/229, 43.23%), *Klebsiella pneumoniae* (35/229, 15.28%), and *Enterococcus* spp. (32/229, 13.97%). Among the 184 Gram-negative bacteria samples, 163 (88.59%) showed a score higher than 2, while 17 (9.24%) scored between 1.7 and 2, and 4 (2.17%) had a score below 1.7 or NP (Table 2). Besides, from the 71 Gram-positive bacterial samples, 27 (38.03%) had a score higher than 2, whereas 20 (28.17%) scored between 1.7 and 2, and 24 (33.8%) samples showed a score lower than 1.7 or NP (Table 2). Interestingly, the performance of MALDI-TOF MS direct identification for *Staphylococcus* spp. was better than that for *Enterococcus* spp. Reliable direct identification was obtained in 14 out of 15 (93.33%) samples belonging to the genus *Staphylococcus*, while just 32 out of 51 (62.75%) from the genus *Enterococcus* were reliably identified. Among the 10 yeast samples found, only 2 were directly identified at the genus level, while 8 samples were NRI (*n* = 4) or NP (*n* = 4). As shown in Figure 1, the turn-around time (TAT) of direct MALDI-TOF MS identification was about 1 h.

**Table 2:** Results of MALDI-TOF MS identification by conventional culture-dependent method and direct method in 265 single*-*microorganism samples.

		No. of samples with MS score by culture method			No. of samples with MS score by direct method			
	Microorganisms	≥2.0	1.7–1.99	<1.7	≥2.0	1.7–1.99	<1.7	NP
Gram-negative bacteria (184)	*Escherichia coli*	97	4		92	7	1	1
	*Klebsiella pneumoniae*	34	2		31	4	1	
	*Klebsiella oxytoca*	4			4			
	*Proteus mirabilis*	7			6	1		
	*Citrobacter koseri*	4			4			
	*Citrobacter freundii*	2			2			
	*Klebsiella aerogenes*	2			1	1		
	*Enterobacter asburiae*	2			2			
	*Enterobacter cloacae*	6			5	1		
	*Morganella morganii*	1			1			
	*Serratia marcescens*	4			4			
	*Pseudomonas aeruginosa*	9	1		7	2	1	
	*Acinetobacter baumannii*	2			2			
	*Acinetobacter pittii*	2			2			
	*Providencia stuartii*	1				1		
Gram-positive bacteria (71)	*Enterococcus faecium*	26	1		10	9	6	2
	*Enterococcus faecalis*	23	1		7	6	8	3
	*Staphylococcus epidermidis*	7			5	1	1	
	*Staphylococcus haemolyticus*	4			3	1		
	*Staphylococcus aureus*	4			2	2		
	*Streptococcus agalactiae*	3	1	1		1	2	2
Yeast (10)	*Candida albicans*	3	1	1		1	2	2
	*Candida glabrata*	2	1			1	1	1
	*Candida tropicalis*	1					1	
	*Candida koseri*	1						1
Total		251	12	2	190	39	24	12

*MALDI-TOF MS, matrix-assisted laser desorption ionisation time-of-flight mass spectrometry; NP, No peaks.*

A total of 22 out of the 1,638 samples had a significant growth of two microorganisms, including 16 samples with bacterial counts ≥5,000 bacteria/μL, and 6 samples with bacterial counts <5,000 bacteria/μL. The 16 samples with counts above the cut-off value were directly identified by the MALDI-TOF MS. Among them, 9 samples corresponded to combined infections by *Candida* and other microorganism, and their results from the direct identification assays were NPR or NP. In addition, 5 samples had a combined growth of *E. coli* + *K. pneumoniae*, 1 sample had a combined growth of *E. coli* + *Proteus mirabilis*, and 1 sample had a combined growth of *Enterobacter cloacae* + *Enterococcus faecalis*. In addition to a score value, the MALDI Biotyper also provides a list of top ten microorganisms with the highest scores. Interestingly, 3 out of these 7 “second pathogens” were found in the top ten list with score value >1.7, and even >2.0.

### VITEK 2 Direct AST Results

Of the 163 Gram-negative bacterial pellets identified at the species level, the first 90, including 72 *Enterobacteriaceae* and 18 non-fermenting Gram-negative bacilli, were collected consecutively and used for direct VITEK 2 AST testing. Unfortunately, due to financial reasons, the remaining 73 pellets did not undergo direct VITEK 2 AST testing. Results were compared to those from the conventional culture-dependent VITEK 2 AST method; the distribution of category agreement and error rates between the two methods are shown in Table 3. A total of 1,296 antimicrobial tests were analyzed in the samples of *Enterobacteriaceae*. There was an overall category agreement rate of 94.83% (1,229/1,296) between the two methods, with minor error rate of 4.17% (54/1,296), major error rate of 0.92% (12/1,296), and very major error rate of 0.08% (1/1,296). The minor errors were mainly found in ampicillin/sulbactam (15.28%), tobramycin (13.89%), cefepime (12.5%), nitrofurantoin (12.5%), imipenem (6.94%), and ceftazidime (5.56%), while the major errors mainly occurred for cefazolin (2.78%), cefepime (2.78%), aztreonam (2.78%), and ertapenem (2.78%). The very major error rate was just found in aztreonam (1.39%). Interestingly, the category agreement rates between the two methods of group U (“urine”) antimicrobial agents, recommended by the CLSI M100 document for UTIs primarily treatment (Clinical and Laboratory Standards Institute [CLSI], 2018b), were very high, 100% for ciprofloxacin, levofloxacin, and sulfamethoxazole/trimethoprim (SMZ/TMP), 97.22% for cefazolin, and 84.72% for nitrofurantoin. A total of 378 antimicrobial tests were analyzed in the samples of the non-fermenting Gram-negative bacilli. There was an overall category agreement of 94.44% (357/378), with rates for minor errors of 2.91% (11/378), major errors of 1.59% (6/378), and very major errors of 1.06% (4/378). The minor errors were mainly found in imipenem (16.67%), meropenem (11.11%), ceftazidime (11.11%), piperacillin (5.56%), cefepime (5.56%), and gentamicin (5.56%), while the major errors mainly occurred for aztreonam (16.67%), piperacillin (5.56%), ceftriaxone (5.56%), and ciprofloxacin (5.56%). The very major errors were found in ceftriaxone (5.56%), imipenem (5.56%), nitrofurantoin (5.56%), and SMZ/TMP (5.56%).

**Table 3:** Categorical agreement and errors for direct AST compared with culture-dependent AST in Gram-negative bacteria.

Microorganisms	Antimicrobial agents	No.	Category agreement		Minor error		Major error		Very major error	
Enterobacteriaceae (*n* = 72)	Ampicillin	72	71	98.61%			1	1.39%		
	Ampicillin/Sulbactam	72	61	84.72%	11	15.28%				
	Piperacillin/Tazobactam	72	71	98.61%	1	1.39%				
	Cefazolin	72	70	97.22%			2	2.78%		
	Cefotetan	72	71	98.61%	1	1.39%				
	Ceftazidime	72	68	94.44%	4	5.56%				
	Ceftriaxone	72	72	100%						
	Cefepime	72	61	84.72%	9	12.5%	2	2.78%		
	Aztreonam	72	67	93.05%	2	2.78%	2	2.78%	1	1.39%
	Ertapenem	72	70	97.22%			2	2.78%		
	Imipenem	72	67	93.05%	5	6.95%				
	Amikacin	72	70	97.22%	2	2.78%				
	Gentamicin	72	71	98.61%			1	1.39%		
	Tobramycin	72	62	86.11%	10	13.89%				
	Ciprofloxacin	72	72	100%						
	Levofloxacin	72	72	100%						
	Nitrofurantoin	72	61	84.72%	9	12.5%	2	2.78%		
	Sulfamethoxazole/Trimethoprim	72	72	100%						
Non-fermenting Gram-negative bacilli (*n* = 18)	Ampicillin	18	18	100%						
	Ampicillin/Sulbactam	18	18	100%						
	Piperacillin	18	16	88.89%	1	5.56%	1	5.56%		
	Piperacillin/Tazobactam	18	18	100%						
	Cefazolin	18	18	100%						
	Cefuroxime	18	18	100%						
	Cefuroxime Axetil	18	18	100%						
	Cefotetan	18	18	100%						
	Ceftazidime	18	16	88.89%	2	11.11%				
	Ceftriaxone	18	16	88.89%			1	5.56%	1	5.56%
	Cefepime	18	17	94.44%	1	5.56%				
	Aztreonam	18	14	77.78%	1	5.56%	3	16.67%		
	Imipenem	18	14	77.78%	3	16.67%			1	5.56%
	Meropenem	18	16	88.89%	2	11.11%				
	Amikacin	18	18	100%						
	Gentamicin	18	17	94.44%	1	5.56%				
	Tobramycin	18	18	100%						
	Ciprofloxacin	18	17	94.44%			1	5.56%		
	Levofloxacin	18	18	100%						
	Nitrofurantoin	18	17	94.44%					1	5.56%
	Sulfamethoxazole/Trimethoprim	18	17	94.44%					1	5.56%

In addition, 27 Gram-positive bacterial pellets identified at the species level, consisting of 10 *Staphylococcus* spp. and 17 *Enterococcus* spp., were used for VITEK 2 direct AST analysis. The distribution of both category agreement and error rates between the direct AST and culture-dependent AST are presented in Table 4. For *Staphylococcus* spp. with a small sample size, the overall categorical agreement between the two methods was 94.38% (151/160), with rates for minor errors of 3.12% (5/160), major errors of 1.87% (3/160), and very major errors of 0.63% (1/160). High minor error rates were detected for ciprofloxacin (20%), levofloxacin (10%), moxifloxacin (10%), and erythromycin (10%); high major error rates were also observed for oxacillin (10%), ciprofloxacin (10%), and vancomycin (10%), and a high very major error rate was found for moxifloxacin (10%). However, it is important to point out that the category agreement rates of group U antimicrobial agents (nitrofurantoin and SMZ/TMP) between the two methods were of 100%. For *Enterococcus* spp., the overall categorical agreement between the two methods was 92.94% (158/170), with rates for minor errors of 2.94% (5/170), and for major errors of 4.12% (7/170). The minor errors were mainly found in ciprofloxacin (5.88%), levofloxacin (5.88%), linezolid (5.88%), vancomycin (5.88%), and nitrofurantoin (5.88%), while the major errors were mainly associated to tetracycline (17.65%), ampicillin (5.88%), linezolid (5.88%), vancomycin (5.88%), and nitrofurantoin (5.88%).

**Table 4:** Categorical agreement and error rates for direct AST compared with culture-dependent AST in Gram-positive bacteria.

Microorganisms	Antimicrobial agents	No.	Category agreement		Minor error		Major error		Very major error	
	Cefoxitin screen	10	10	100%						
*Staphylococcus* ssp. (*n* = 10)	Benzylpenicillin	10	10	100%						
	Oxacillin	10	9	90%			1	10%		
	Gentamicin	10	10	100%						
	Ciprofloxacin	10	7	70%	2	20%	1	10%		
	Levofloxacin	10	9	90%	1	10%				
	Moxifloxacin	10	8	80%	1	10%			1	10%
	Erythromycin	10	9	90%	1	10%				
	Clindamycin	10	10	100%						
	Quinupristin-dalfopristin	10	10	100%						
	Linezolid	10	10	100%						
	Vancomycin	10	9	90%			1	10%		
	Tetracycline	10	10	100%						
	Nitrofurantoin	10	10	100%						
	Rifampicin	10	10	100%						
	Trimethoprim/Sulfamethoxazole	10	10	100%						
*Enterococcus* ssp. (*n* = 17)	Benzylpenicillin	17	17	100%						
	Ampicillin	17	16	94.12%			1	5.88%		
	Ciprofloxacin	17	16	94.12%	1	5.88%				
	Levofloxacin	17	16	94.12%	1	5.88%				
	Rifampicin	17	17	100%						
	Quinupristin-dalfopristin	17	17	100%						
	Linezolid	17	15	88.24%	1	5.88%	1	5.88%		
	Vancomycin	17	15	88.24%	1	5.88%	1	5.88%		
	Tetracycline	17	14	82.35%			3	17.65%		
	Nitrofurantoin	17	15	88.24%	1	5.88%	1	5.88%		

## Discussion

In recent years, MALDI-TOF MS-based combination methods have demonstrated good stability, accuracy, and speed in the direct identification and AST of pathogens present in clinical specimens, such as blood and sterile body fluids (Tian et al., 2016; Di Gaudio et al., 2018; Wu et al., 2019). In our previous study, we developed a fast and easy lysis-centrifugation-wash protocol to prepare bacterial pellets from blood cultures positive for the presence of microbial pathogens, that can be used for direct pathogen identification by MALDI-TOF MS and for direct AST using the VITEK 2 system (Pan et al., 2018). It allows clinically helpful results to be reported from days to hours after a positive blood culture system alarm. However, blood and sterile body fluids need to be injected into blood culture bottles and incubated for several, even dozens of, hours to get a positive alarm, while urine, especially morning urine, is a sample that is naturally incubated inside the bladder. This makes it possible to identify pathogens directly from the urine of patients presenting UTIs, without spending time on incubation.

The purpose of this study was to develop a MALDI-TOF MS-based workflow to shorten the time needed for microbial identification and for obtaining AST results for UTIs. The performance and optimisation of a combination of flow cytometry and MALDI-TOF MS for screening and direct identification of pathogens in urine has been reported previously; several other procedures have been described as well. Initially, Ferreira et al. (2010) established a direct identification method based on flow cytometry screening and MALDI-TOF MS. Sánchez-Juanes et al. (2014) added a pre-treatment step with SDS to this, to lyse cells and release microorganisms, in order to enhance the method’s sensitivity. Veron et al. (2015) performed a comparison between three different methods, and found that the previous 5 h culturing and dual-filtration methods provided the better identification results. Kitagawa et al. (2018) added ultrasonication for 10 min at the beginning of the procedure, followed by centrifugation, to disperse bacterial cell aggregation. Íñigo et al. (2016) established that using a 2 mL volume of urine was optimal for sample manipulation in Eppendorf tubes and centrifuges. While Zboromyrska et al. used 10 mL of urine with bacterial counts of ≥5,000 bacteria/μL for MALDI-TOF MS direct identification and obtained high scores (Zboromyrska et al., 2016). Moreover, these parameters were confirmed by a multicentre study that followed soon after (Zboromyrska et al., 2018).

In this study, we suggest the usage of a differential centrifugation method to prepare bacterial pellets for analysis from a starting urine volume of 30 mL (10 mL for screening, 10 mL for identification, and 10 mL for AST). This is easily achievable as urine is one of the most easily collected clinical samples and 15 mL centrifuge tubes and centrifuges are routinely available in clinical microbiology laboratories. We also confirmed the accuracy of the pre-established cut-off value of ≥5,000 bacteria/μL for bacterial counts as a sample selection criterium for direct identification by MALDI-TOF MS. In this study, flow cytometry analysis results showed that 18.74% (307/1,638) of urine samples were positive for infections and should be used for further direct identification tests, and that the omission ratio of positive culture samples was 6.35% (104/1,638).

Overall, a reliable direct identification was obtained in 86.42% of the single-microorganism samples, in accordance with previous studies (Zboromyrska et al., 2016). Until now, reliable identification of Gram-negative bacteria has proven to be more efficient than that of Gram-positive bacteria, with 97.83% versus 66.2% bacteria identified reliably, respectively. Moreover, the performance of MALDI-TOF MS direct identification for *Staphylococcus* spp. (93.33%) was better than that for *Enterococcus* spp. (62.75%). This may be partly explained by the unsuccessful removal of urine cells during sample processing by the differential centrifugation method to prepare pellets of *Enterococcus* spp. In some urine samples with significant growth of *Enterococcus* spp., the direct identification results were either NRI or NP, so we prepared a smear using these pellets. After Gram-staining, a certain amount of epithelial cells and leukocytes was found to still be present in the smear (data not shown). Poor performance was also observed in the identification of yeasts; only 2 out of 10 samples were directly identified at the genus-level, in accordance with a study by Burillo and better than results from Íñigo (Burillo et al., 2014; Íñigo et al., 2016). Therefore, direct identification of yeasts from urine samples still constitutes a challenge for achieving quick and efficient diagnosis.

Compared to the conventional culture-dependent VITEK 2 AST, the overall category agreement of the direct AST was 94.56%, with 94.83% for *Enterobacteriaceae*, 94.44% for the non-fermenting Gram-negative bacilli, 94.38% for *Staphylococcus* spp., and 92.94% for *Enterococcus* spp. With respect to *Enterobacteriaceae*, the most common causative agent of UTIs, a very good concordance was observed between the two AST methods for the group U or first-line antimicrobial agents, which yielded essential agreement rates of 100% for ciprofloxacin, levofloxacin, SMZ/TMP, and ceftriaxone, and 97.22% for cefazolin. However, an unacceptable minor error rate (12.5%) was obtained for nitrofurantoin.

For *Staphylococcus* spp. and *Enterococcus* spp., the error rates of several antimicrobial agents exceeded CLSI recommendations (Clinical and Laboratory Standards Institute [CLSI], 2018a). These results may be due to the small sample size obtained for these bacteria in our study (*n* = 10 and *n* = 17, respectively). The small number of *Staphylococcus* spp. and *Enterococcus* spp. samples obtained for direct AST analysis may be because of the high bacterial count cut-off value used in this study, which is more frequently achieved by Gram-negative bacteria than by Gram-positive bacteria (Monsen and Rydén, 2015; Zboromyrska et al., 2016). Moreover, it is important to point out that the category agreement rates of nitrofurantoin and SMZ/TMP against *Staphylococcus* spp. were 100%.

The minor and major errors calculated for vancomycin and linezolid may be due to urine sample contamination with a small amount of Gram-negative bacilli, whose growth on plates may have been covered by the growth of Gram-positive bacteria. The number of these Gram-negative bacilli could have been so small that they were not observed on plates nor directly detected by MALDI-TOF MS. Wang et al. analyzed urine specimens containing different *E. coli* and *Enterococcus faecium* proportion ratios by MALDI-TOF MS, and showed that *E. coli* and *Enterococcus faecium* were simultaneously detected if the mixture ratio was 1:1 or 1:2, but only the dominant pathogen was detected at mixture ratio of 1:9 (Wang et al., 2013).

Our urine processing methodology includes differential centrifugation and two washes to enrich the bacterial fraction for MALDI-TOF MS and AST assays. The entire process can deliver identification results within 1 h. For the most common pathogens of UTIs, *Enterobacteriaceae*, it usually takes 6–8 h to yield a final AST report using the VITEK 2 system. Therefore, our MALDI-TOF MS-based combined protocol could provide reliable “same day” reports including identification and ASTs for *Enterobacteriaceae*, which are important for a quick treatment and to improve clinical outcomes of UTIs. Patients with uncontrolled UTIs are likely to develop secondary blood stream infections, even urosepsis, especially those patients with obstructive uropathy and wearing invasive devices, such as catheters (Dreger et al., 2015). Early identification of pathogens responsible for UTIs and early treatments based on results from fast AST are the best tools for urosepsis prevention (Wagenlehner et al., 2017). The “same day” report achieved through our protocol may result in early pathogenic diagnoses and early treatment of UTIs, reducing the incidence of urinary sepsis.

There are nevertheless some limitations to our method that should be considered for future applications, such as the high starting volume of urine, the need for bacterial counts ≥ 5,000 bacteria/μL, and the exclusivity of application to monomicrobial infections. Additionally, our study has been limited by the reduced number of samples analyzed and the small number of samples found for some bacterial groups, such as *Staphylococcus*, *Enterococcus*, and non-fermenting Gram-negative bacilli.

In conclusion, combining flow cytometry, MALDI-TOF MS, and the VITEK 2 system allows the reliable identification and AST of UTI pathogens directly from urine samples, especially in cases of Gram-negative bacterial infections.

## Data Availability

All datasets generated for this study are included in the manuscript.

## Ethics Statement

This study was reviewed and approved by the research ethics committee of Qilu Hospital of Shandong University (protocol KYLL-2019-277). The participants provided a written informed consent when their urine samples were included in the study.

## Author Contributions

CW, YZ, ES, WL, and HP designed the experiments. WL, YW, and HP performed the experiments. YL and CL helped perform the experiments. WL and CW analyzed the data. XZ and LD helped analyze the data. WL and ES wrote the manuscript. All the authors read and approved the final version of the manuscript.

## Conflict of Interest Statement

The authors declare that the research was conducted in the absence of any commercial or financial relationships that could be construed as a potential conflict of interest.
